# Allergy in pathogenesis of Eustachian Tube Dysfunction

**DOI:** 10.1016/j.waojou.2023.100860

**Published:** 2024-01-05

**Authors:** Xuan Yu, Huimin Zhang, Shimin Zong, Hongjun Xiao

**Affiliations:** Department of Otorhinolaryngology, Union Hospital, Tongji Medical College, Huazhong University of Science and Technology, Wuhan 430022, China

**Keywords:** Eustachian tube dysfunction, Allergy, Th2 cytokines, Surfactant, Anti-allergic therapy

## Abstract

Eustachian tube dysfunction (ETD) is a condition where the Eustachian tube (ET) fails to function normally, resulting in symptoms such as aural fullness, tinnitus, autophony, and hearing loss. ETD can further lead to middle ear diseases such as otitis media effusion and adhesive otitis media, which is becoming more common in the field of otology. Although the pathogenesis of ETD remains unclear, recent animal studies and clinical experiments have found allergic reactions and allergic diseases are closely related to the occurrence of ETD. As the mucosa of the ET is continuous with that of the nasopharynx and tympanic cavity, it is reasonable to assume that the immunological basis of the ET itself is similar to that of respiratory allergic diseases. However, due to the special anatomical location and complex pathogenesis of the ET, there is still no unified diagnostic gold standard. Additionally, there is an ongoing debate regarding whether ETD can be classified as a distinct disease or even an allergic disease. Furthermore, the effectiveness of anti-allergic therapy in patients with ETD is yet to be fully understood. Therefore, this review elaborates on the possible mechanisms of allergic reactions in the occurrence and development of ETD, and explores the potential role of anti-allergic therapy in managing this condition, in order to provide new insights into the pathogenesis and prevention of ETD.

The Eustachian tube (ET) is a tubular structure that connects the nasopharynx and the middle ear cavity. Its physiological functions include ventilation and regulation of middle ear pressure, clearance of middle ear mucous secretions, protection of the middle ear against sounds, and prevention of retrograde infection from the nasopharynx.^1^ Eustachian tube dysfunction (ETD) is a syndrome caused by 1 or more dysfunctions of the ET, which often suggests a constellation of symptoms and signs associated with impaired Eustachian tube function.^2^ Research has shown that the overall prevalence rate of ETD in adults in the United States is approximately 4.6%,^3^ and it often clinically presents as symptoms such as aural fullness, tinnitus, autophony and hearing loss, which severely affects which severely affect patient quality of life. Due to the unique anatomical position, complex pathophysiological mechanisms, and non-specific symptoms of ETD, there are currently no unified diagnostic criteria.^2^^,^^4^ The main diagnostic method at present is based on clinical symptoms and signs, combined with objective examinations, to improve the accuracy of diagnosis of ETD.

In the 2015 International Consensus Statement on Eustachian Tube Dysfunction, ETD is classified into acute ETD (transient with symptoms and signs for less than 3 months) and chronic ETD (symptoms and signs for more than 3 months), with the latter further divided into 3 subtypes: baro-challenge-induced ETD, patulous ETD, and dilatory ETD.^2^ Dilatory ETD is the most common type of ETD in clinical practice,^5^ and is also one of the important reasons for chronic suppurative otitis media that is prolonged or repeatedly acute. It may be related to functional obstruction, dynamic dysfunction(muscular failure), and anatomical obstruction.^2^ Therefore, this obstructive ETD is the focus of this review.

Currently, multiple studies have shown that ETD is associated with allergic reactions. The possible mechanisms of allergic reactions in ETD include: (1) inflammation mediators and mucus damage mucociliary clearance system function in the ET mucosa; (2) self-inflammation of the ET leads to mechanical obstruction caused by mucosal inflammation and swelling; (3) inflammatory mediators reduce surfactant in the ET, impairing its active opening function; (4) the spread of inflammation from adjacent areas (such as the nasopharynx) to the ET; and (5) late-phase allergic reactions may also participate in the development of ETD.

However, there is still controversy regarding the relationship between ETD and allergic reactions. Firstly, the specific pathophysiological mechanisms of ETD are not yet fully understood. Although ETD is considered to be one of the important causes of diseases such as otitis media with effusion (OME) and suppurative otitis media, a large amount of research is still focused on the role of allergic reactions in the development of OME, and there is relatively little literature on the association between allergy and simple ETD. Secondly, it remains uncertain whether chronic dilatory ETD is a continuation of the same pathology that causes acute ETD, or if other pathological mechanisms are responsible for these symptoms.^2^ Therefore, this article will review the research progress on possible allergic mechanisms in ETD and aims to provide a reference for future studies.

## Possible mechanisms of allergic responses in the occurrence and development of ETD

### The immunological basis of the ET

#### Unified airway concept

The concept of a unified airway describes the upper and lower airways as a single, interconnected unit, meaning that stimulation in one area can lead to inflammation throughout the entire system.^6^ Specifically, the respiratory mucosa of the middle ear, nose and sinuses, and lower respiratory tract are structurally and physiologically uniform, consisting of pseudostratified columnar epithelium with goblet cells that secrete mucus and participate in active transport of mucus and particles.^6^ Research has shown that the inflammatory mediators released in respiratory diseases are the same throughout the entire respiratory system, including T-cell cytokines such as interleukins IL-4, IL-5, and IL-13, as well as eosinophilic cells.^7^ Additionally, the middle ear mucosa, like other parts of the upper respiratory epithelium, evolved from the ectoderm and has been shown in animal studies to have the same immune reactivity to antigens as the nasal cavity, sinuses, and bronchi.^8^ Nguyen et al. found parallel allergic inflammation in the ET and nasopharynx or upper airway in patients with atopic otitis media, further supporting the view that the middle ear cavity is a component of the unified airway.^9^ Therefore, as a conduit structure connecting the nasopharynx and middle ear cavity, the mucosa of ET is continuous with that of the nasopharynx and middle ear, and it is reasonable to assume that the ET itself has an immunological basis similar to that of respiratory allergic diseases.

#### The immunomodulatory role of cytokines in the pathogenesis of ETD

The allergic responses begin with the sensitization phase, which occurs when the body is first exposed to an allergen without showing clinical symptoms. When the allergen is encountered again, it triggers allergic reactions, resulting in clinical symptoms. During this process, a large number of allergic mediators are produced, including various cytokines and chemokines. The production and accumulation of these allergic mediators can trigger and sustain the allergic response.

##### T helper 2 (Th2) cytokines

Allergic reactions are type I hypersensitivity reactions mediated by IgE and driven by Th2 cytokines response.^10^ A large body of research has confirmed the role of Th2 cytokines in allergic immunity, but there are no literature reports directly detecting allergic-related immune factors in the ET. Therefore, if the presence of these Th2 cytokines could be detected in ETD, it would more strongly support the view that allergy is a pathogenic factor in ETD.

###### IL-4, IL-5

IL-4 and IL-5 are 2 important Th2 cytokines that are involved in IgE-mediated allergic reactions. Studies have shown that IL-4 mainly promotes the differentiation of Th0 cells (undifferentiated T helper cells) into Th2 cells by activating the transcription factor STAT6 and inducing the expression of GATA3, promoting the expression of IL-4 and IL-5, thus strengthening the positive feedback loop of Th2 cell differentiation (Supplemental Figure 1A). In short, IL-4 is responsible for initiating allergic reactions and producing IgE.^11^ IL-5, as a colony-stimulating factor for eosinophils, recruits and activates eosinophils, promoting their differentiation and maturation, and therefore plays an important role in the late-phase allergic reactions.^12^

Although IL-4 and IL-5 have not been directly detected in the ET, a study by Pollock et al. found that in a rat model of OME induced by systemic sensitization with ovalbumin (OVA) and stimulated in the middle ear, the ET ventilation and clearance function remained relatively stable in the group that pre-treated with IL-4 antagonists, with normal mucociliary function, compared with the control group that did not use antagonists.^13^ In contrast, the group pre-treated with IL-5 antagonist did not show significant increase in eosinophils, but exhibited more severe inflammation and edema, with impaired mucociliary function in the ET. The study concluded that IL-4 antagonist administered via the middle ear can effectively restore the ventilation and clearance function of the ET in sensitized animals, and prevent the occurrence of ETD. In addition, IL-4 antagonist has a stronger effect than IL-5 antagonist in alleviating allergy-mediated ETD. These also indirectly confirm the presence of IL-4 and IL-5 in the ET inflammation and their involvement in the allergic reactions process of ETD.

In addition, the unified airway concept mentioned earlier provides a theoretical basis for indirectly reflecting the immune environment within the ET. In recent years, a large number of animal studies have found that in the allergic OME model, the expression levels of eosinophils, IL-4, IL-5 and other related immune factors in the middle ear mucosa and middle ear bone marrow cavity are significantly higher than those in the control group.^14^, ^15^, ^16^, ^17^ Most of these studies observed similar results: after modeling, the ET mucosa thickened, the ciliated epithelium was disordered and shed, and serum IgE expression increased. Based on this, Shi et al evaluated the degree of mucosal changes in the ET, middle ear cavity, and tympanic membrane tension zone, and found that the changes in the opening at the entrance of the ET were the most obvious, suggesting that allergic reactions may occur earlier in the ET and induce changes in middle ear inflammation.^17^ This provides indirect evidence of the role of allergic reactions in ETD and its complications. In addition, Wright et al. found that IL-5 mRNA expression levels were elevated in the middle ear mucosa of children with persistent OME.^18^

###### IL-13

IL-13 is a key effector cytokine in allergic inflammation, which can synergize with IL-4 to promote B cell differentiation into plasma cells and synthesize IgE. At the same time, it rapidly synthesizes specific IgE when the allergen is re-stimulated. Previous studies have shown that IL-13 plays an important role in inducing strong airway hyperresponsiveness and pathological changes associated with allergic airway diseases.^19^ However, there is little literature on determining the presence and role of this factor in OME or ETD. A recent study found that the expression of IL-5 and IL-13 in the middle ear mucosa of allergic OME mice induced by intraperitoneal injection of OVA combined with nasal stimulation was significantly higher than that in the control group. Although the study did not evaluate the ET patency or function of the allergic group, they believed that allergy is likely to affect ET function.^20^

In addition, IL-13 is also considered to play an important role in inducing airway mucosal goblet cell hyperplasia and mucus production. Studies have shown that IL-13 induces goblet cell metaplasia by activating the epidermal growth factor cascade, resulting in increased expression of mucin^21^ (Supplemental Figure 1A). Mucin is a key factor in maintaining MCC function in the middle ear and ET, so IL-13 may be involved in the allergic mechanism of ETD by regulating mucin secretion.

##### IL-8

Currently, there is no literature reporting direct detection of IL-8 in ETD. However, it should be noted that Kerschner et al. confirmed through RT-PCR that IL-8 can significantly upregulate the expression of middle ear mucin gene MUC5AC.^22^ The ET can express equivalent amounts of MUC5B and MUC5AC, which suggests that IL-8 may also affect ETD by causing mucin disorder^23^ (Supplemental Figure 1A). In addition, a large number of studies have shown that early middle ear effusion in OME may be directly related to high expression of IL-8.^24^ IL-8 can bind to its receptor, causing cell deformation, degranulation, release of lysosomes and peroxides, while enhancing neutrophil expression of adhesion molecules and increasing capillary permeability. This may lead to the ET mucosal edema and damage to mucociliary clearance function, resulting in ETD.

#### Inflammation mediators and mucus damage mucociliary clearance (MCC) system function in the ET mucosa

The surface of the ET is covered by a pseudostratified ciliated columnar epithelium, and the ciliated cells, together with the overlying mucus blanket, form the mucociliary transport system.^25^ Studies have found that the cilia movement direction, which is directed from the middle ear cavity towards the nasopharynx, allows the ET to expel secretions and pathogens towards the nasopharynx.^26^ In addition, the normal functioning of the mucociliary system in the ET can prevent excess secretions from accumulating in the ear. However, type I hypersensitivity reactions can affect ciliary movement and damage the mucosal barrier function of the respiratory tract. Tanaka et al sensitized guinea pigs with ovalbumin and found that systemic and local allergic reactions accelerated ciliary activity, but may affect the ET mucus blanket and induce mucociliary dysfunction.^26^ Hardy et al confirmed that allergen stimulation in the ear can induce inflammation in the middle ear and the ET mucosal system, impairing the clearance ability of the mucociliary system and leading to ETD.^27^ Further animal experiments conducted by Liu et al successfully induced type I hypersensitivity reactions in the ET of rats through sensitization of allergens in the peritoneal cavity and stimulation in the middle ear.^14^ The results showed that the experimental group had swelling and disordered arrangement of ciliated epithelium in the ET drum segment, as well as partial loss of cilia and MCC dysfunction, leading to acute otitis media with effusion (AOM).

Mucin is an important glycoprotein in the mucociliary transport system of the middle ear and ET, which is the main component of middle ear effusion and plays an important role in maintaining the function of the MCC system. However, excessive production of mucin and changes in mucin types can disrupt the normal MCC function of the ET, ultimately leading to ETD and even middle ear disease.^28^ Studies have shown that Th2-related cytokines such as IL-4, IL-5, and IL-13 can participate in the expression and secretion of mucin genes MUC5AC and MUC5B,^29^, ^30^, ^31^ further indicating the role of allergy in the occurrence and development of ETD.

#### Self-inflammation of the ET leads to mechanical obstruction caused by mucosal inflammation and swelling

Histological studies have shown that the levels of allergic cell factors such as IL-4, IL-5, and eosinophils are elevated at both ends of the ET,^32^ indicating the possibility of allergic inflammation in the ET. Therefore, in addition to the mechanisms mentioned earlier, the inherent hyperreactive inflammation of ET itself may cause intrinsic venous engorgement and hypersecretion of mucus, leading to mechanical obstruction and isolation of the middle ear space, affecting gas exchange.^33^ If left untreated, prolonged ET obstruction can result in chronic inflammation of the middle ear, which can lead to epithelial metaplasia, increased glandular activities of goblet cells, and an inability to clear middle ear fluid (Supplemental Figure 1B).^33^

#### Inflammatory mediators reduce the ET surfactant, impairing its active opening function

The surfactant in the ET is made up of lipids, phospholipids, and four surfactant proteins (SP-A through SP-D), which play a crucial role in altering the rheological properties of the ET mucus, improving the efficiency of the mucociliary system, and enhancing immune function.^34^^,^^35^ Research has shown that the surfactant can improve the rheological properties of ET mucus and has an anti-adhesive function.^35^^,^^36^ At the same time, the surfactant can also increase the ciliary beat frequency by reducing the depth of mucus, effectively promoting the discharge of mucus in the ET.^34^ If the ET becomes inflamed or suffers from inflammation, the increase in its opening pressure can lead to impaired ET active opening function, resulting in ETD (Supplemental Figure 1C). Previous animal studies have demonstrated that surfactant treatment can improve ciliary function in the damaged ET and significantly reduce middle ear fluid in guinea pigs with OME, compared to the control group.^37^^,^^38^ Therefore, it can be inferred that the ET surfactant has a positive therapeutic effect on ETD. It is worth noting that surfactant protein D among the four surfactant proteins seems to have no contribution to surface tension, but it helps to regulate the innate immune response and homeostasis of the ET mucosa.^39^

In summary, the generation and accumulation of inflammatory mediators in ET can lead to a reduction in surfactant, which can in turn cause ETD by affecting the function of the ET. Although the impact of allergic reactions in ET on surfactant has not been studied, we look forward to further exploration in this area in the future.

### The spread of inflammation from adjacent areas (such as the nasopharynx) to ET

#### Allergic rhinitis (AR)

Although a large body of literature suggests an association between AR and OME or ETD in children, there is currently no clear causal relationship. A common view is that chronic ETD is caused by long-term nasal inflammation, especially nasal allergy. Fu et al found that compared with the non-AR control group (healthy adults), the 2 groups of ET morphology and middle ear function in 100 AR patients had statistically significant differences (two ET morphology (χ^2^ = 11.538, *p* < 0.01), tympanogram curve (χ^2^ = 14.519, *p* < 0.01)), suggesting that AR can affect the morphology of the ET and middle ear function, and the more severe the AR reaction, the more likely it is to cause ETD.^40^ The research results of Ma et al also confirmed this view.^41^ Another study found through analysis of the 2011–2012 US National Health and Nutrition Examination Survey database that compared with patients without ETD (bilateral type A tympanogram, *p* = 0.039), patients with ETD had a 1.71 times higher risk of concurrent allergy, and AR was significantly correlated with ETD.^42^ However, a prospective study found no difference in ET function among AR patients who underwent nasal provocation testing. Additionally, there was no difference in the incidence of AR between children with OME with and without AR, suggesting that AR is not directly related to the occurrence of OME.^43^

Currently, research suggests that there are two mechanisms by which AR may lead to ETD (Supplemental Figure 1D). On the one hand, allergens inhaled by AR patients may deposit on the surface of the nasal and nasopharyngeal mucosa, activating mast cells containing IgE, leading to the release of inflammatory mediators, increasing local vascular permeability and mucus production, ultimately resulting in obstruction of the pharyngeal orifice of the ET and impairment of Eustachian tube function.^44^ On the other hand, inflammatory mediators in the nasal mucosa may also directly spread to the ET, leading to ciliary clearance dysfunction and subsequent ETD.^45^ Shi et al successfully constructed an animal model of OME by using OVA systemic sensitization and nasal stimulation method. From the tissue structure, they found that the mucosal tissue changes in the ET and the middle ear near the ET were the most obvious in allergic reactions.^17^ This also indicates that inflammation in the nasopharynx can spread to the ET and cause middle ear disease through the ET.

#### Adenoidal hypertrophy (AH)

The adenoid is also considered to have a potential connection with ETD, especially in its pathological condition. The general view is that AH can cause mechanical obstruction of the ET or affect its ventilation and drainage function, leading to ETD (Supplemental Figure 1D). However, not all AH patients will develop OME, and studies has shown that when the adenoids are in close contact with the torus tubarius, adenoidectomy can significantly improve ETD, while the size of the adenoids is completely unrelated.^46^^,^^47^ This suggests that local inflammatory mediators or the presence of biofilms may be another mechanism leading to AH induces OME or ETD.^48^ When AH is present, the lymphocyte regulatory function of the adenoid is lost, and various inflammatory cytokines are passively released, increasing vascular permeability and exacerbating damage to the mucosa of the middle ear and ET.^49^ Studies have shown that children with OME have higher levels of histamine and subepithelial mast cells in their adenoid tissue than those without OME.^50^ In addition, a review mentioned that the adenoid and ET may be potential target organs for allergic inflammation, leading to long-term dysfunction of the ET and causing bacterial or viral reflux and middle ear infections.^28^

### The role of late-phase allergic reactions in the development of ETD

Late-phase reactions is the late stage of allergic reactions, also known as the late-phase allergic reactions (LPR). LPR usually occurs 8–24 h after the early-phase allergic reactions, lasting for 24–48 h, or even several weeks, leading to excessive allergic reactions in affected tissues.^51^ Hardy et al has confirmed that sensitized rats injected with OVA through the tympanic membrane developed ETD and OME, which lasted for at least 32 h after stimulation, suggesting that these changes were secondary to LPR.^27^ Therefore, it is believed that LPR can induce ET epithelial inflammation, leading to ET blockage, middle ear ventilation and MCC function disorders, and even OME. Pollock et al. used IL-4 and IL-5 antagonists to pretreat sensitized rats injected with OVA through the tympanic membrane, confirming that IL-4 antagonists were effective in treating ETD and OME caused by LPR.^13^ These studies suggest that LPR can further exacerbate allergic reactions in the ET and may play a role in recurrent acute ETD or chronic ETD and chronic middle ear disease, which should be given attention in future research.

## Application of anti-allergic therapy in the development of ETD

Although many studies have confirmed the association between allergic reactions and ETD, the efficacy of anti-allergic therapy for ETD has been questioned.^52^ Current anti-allergic therapy includes antihistamines, intranasal corticosteroids (INCs), immunotherapy, etc.

### Histamine and antihistamines

Histamine is the main inflammatory mediator in early allergic reactions, which can cause high secretion of mucosal glands and local vasodilation.^10^ Antihistamines are believed to effectively inhibit early phase allergic reactions, reduce mucosal congestion, and alleviate histamine-driven symptoms. Therefore, it seems reasonable to use antihistamines to regulate the inflammatory response in ETD. However, there is still controversy over whether antihistamines play a role in the treatment or prevention of ETD.

The evidence supporting the role of histamine or anti-histamine drugs in the development of ETD is mainly derived from animal experiment. Downs et al. sensitized rats through intratympanic and intranasal histamine stimulation and found that histamine could affect rat ET ventilation and ciliary clearance function, leading to acute ETD.^53^ Ebert et al. successfully induced acute ETD in rats through intranasal histamine infusion, with symptoms similar to those observed in the previous study.^54^ A recent randomized, double-blind study tested the effects of intranasal histamine and placebo stimulation on middle ear pressure balance and gas exchange performance, and found that histamine could cause inflammation of the local mucosa and the distal mucosa, including the ET mucosa, thereby affecting ET opening and causing ETD.^55^ In addition, many studies have shown that the use of intra-aural antihistamines in sensitized rat models can reduce the accumulation of middle ear fluid, improve OME symptoms, and may be effective in treating ETD caused by allergies.^56^, ^57^, ^58^

However, most studies indicate that antihistamines are not effective in treating ETD. On the one hand, this may be because the anticholinergic effects of antihistamines can reduce ciliary rhythm frequency and increase mucus viscosity,^59^ interfering with ET drainage or absorption and causing dysfunction of the ET epithelium. On the other hand, antihistamines can also affect glandular cell secretion function, causing additional side effects such as dry mouth, constipation, bloating, and tension. Although second-generation antihistamines have less side effects compared to first-generation ones, which have anticholinergic activity,^60^ first-generation antihistamines are still widely used, and there is limited literature on the improvement of ETD with second-generation antihistamines, which is one of the reasons for controversy. A randomized controlled trial by Chonmaitree et al found that the use of antihistamines (chlorpheniramine maleate) during an acute episode of OME did not improve outcomes and instead prolonged the duration of middle ear fluid.^61^ As ET is generally used for drainage of middle ear effusion, they speculated that antihistamines may inhibit ET function during acute inflammation. Griffin et al. conducted a meta-analysis and systematic review in 2006 and updated it in 2011, finding that the use of antihistamines, decongestants, or a combination of both had no benefit in preventing or treating OME, and the incidence of side effects was about 10%.^60^^,^^62^ Subsequently, a meta-analysis by Roditi suggested that INCs and antihistamines were both ineffective in achieving long-term improvement in OME.^63^ Therefore, antihistamines have not shown long-term benefits in either ETD or OME, and are not recommended as clinical treatment in multiple guidelines and studies. However, the emergence of second-generation antihistamines and new-generation drugs is worth further research in the future.

### Intranasal corticosteroids

It is well known that corticosteroids have significant anti-inflammatory and anti-edema effects. Studies have reported that corticosteroid can improve ETD by enhancing the secretion of the ET surfactant.^64^ Although INCs and antibiotics have become the most commonly prescribed drugs in various specialties for acute ETD patients,^65^ the treatment plan for using INCs to treat ETD is still unclear, and research in this area is limited, with most studies focusing on treating OME.

Wu et al. conducted a small retrospective cohort study to evaluate the effectiveness of a novel fluticasone propionate exhalation delivery system (Xhance nasal spray, referred to as EDS-FLU) in improving symptoms of ETD.^66^ The study results showed that after one month of treatment, most patients (approximately 79%) experienced improvement in symptoms, with 36% of patients achieving a normal level on the ETDQ-7 scores. This favorable treatment outcome may be attributed to the ability of EDS-FLU, compared to other nasal sprays, to deliver the medication more effectively to the nasopharynx and ET orifices. It should be noted, however, that this study has limitations in terms of sample size, exclusion of comorbidities, and the use of tympanometry or audiometry before and after treatment. Other studies do not seem to support the efficacy of INCs in treating ETD. Gluth et al. conducted a prospective RCT that included adult and relatively older children with ETD and evaluated the effectiveness of INCs in treating ETD based on tympanogram and related symptoms including OME and middle ear negative pressure.^67^ The results showed that the use of INCs in treating ETD was not supported. A systematic review by Llewellyn et al. also indicated that INCs do not improve symptoms and middle ear function in patients with middle ear fluid and/or negative middle ear pressure.^68^ The latest systematic review comparing the efficacy of existing drug treatments for obstructive ETD in adults showed that INCs did not significantly improve symptoms in patients with chronic ETD (only effective in improving symptoms in 11%–18% of chronic ETD patients).^69^ This suggests that INCs are not an effective treatment option for chronic ETD patients.

Although OME is not solely caused by ETD, the treatment of OME has a positive impact on ETD. A small study by El-Anwar et al. compared the effectiveness of intranasal mometasone furoate spray, oral prednisolone, and saline nasal spray in treating OME.^70^ The results showed that systemic or intranasal corticosteroid treatment was significantly more effective than saline nasal spray (*p* < 0.001), and INCs were effective in treating OME. However, most studies suggest that INCs are not helpful in improving ETD or OME. Williamson et al. conducted a randomized double-blind placebo-controlled trial to evaluate the efficacy of nasal steroid in children with bilateral OME.^71^ The results indicated that topical corticosteroids are unlikely to be an effective method for treating OME in general. Berkman's systematic review compared the treatment strategies for OME in terms of efficacy and effectiveness and concluded that INCs did not show a difference in cure rate when used alone or in combination with antibiotics.^72^ Multiple studies have also shown that INCs have no long-term benefits for otitis media effusion.^64^^,^^73^ In addition, clinical practice guideline for OME developed by the American Academy of Otolaryngology-Head and Neck Surgery Foundation, the American Academy of Family Physicians, and the American Academy of Pediatrics also recommend against using INCs in OME.^74^

#### Treatment of comorbidities with individual or combined use of INCs

Considering the complexity of the development process of ETD, there is currently insufficient evidence to determine the effectiveness of combination therapy for OME or ETD.^72^ At the same time, there is still no consensus on the efficacy of antihistamines, or INCs, or combination therapy for ETD with significant allergic diseases (such as AR or AH) or simple ETD.

Treatment of ETD associated with allergic diseases using either individual INCs or combination therapy: Crowson et al. conducted a retrospective case study on ETD patients with combined OME treated with intranasal fluticasone, aiming to determine whether this treatment can prevent the need for tympanostomy tube placement in children with ETD.^75^ The results showed that INCs significantly reduced the number of ETD patients requiring tympanostomy, while controlling for gender, race, socioeconomic indicators, and the presence of CLP or Down syndrome. However, this trial did not involve expert evaluations of the subjects or consider other factors contributing to ETD, such as allergic diseases and gastroesophageal reflux disease, which may have influenced the accuracy of the data and results. Ma et al. conducted a prospective controlled cross-sectional study that included 59 patients with AR who were treated with mometasone furoate nasal spray and oral loratadine for one month.^41^ The study evaluated the nasal and ET conditions before and after treatment and found that the AR patients' visual analogue scale (VAS) scores, nasal endoscopic scores, and Eustachian Tube Dysfunction Questionnaire-7 (ETDQ-7) scores all significantly decreased (*p* < 0.0001). This suggests that the combination of INCs and antihistamines can significantly improve ET function as nasal symptoms improve. This result is likely due to the relief of AR symptoms, which improves ETD symptoms. Although few studies have distinguished the therapeutic effects of using INCs to treat patients with AR combined with ETD for the treatment of rhinitis and ETD, *the Chinese Consensus on Eustachian Tube Dysfunction*^1^ suggests that INCs can be given to ETD patients with AR. Studies have also found that combination therapy appears to be effective in treating ETD with AH. A recent study evaluated the efficacy of 3-month intranasal Azelastine-Fluticasone dipropionate combination in treating children with AH and ETD, and found that combination therapy was significantly effective in reducing adenoid tissue and improving ETD after three months of treatment.^76^

In addition, several studies have shown that INCs are beneficial in treating OME patients with AH.^77^^,^^78^ Overall, there is currently no consensus on the use of INCs for the treatment of ETD or OME. Future research should further investigate the duration, dosage, and appropriate population for INCs. At the same time, in future experimental designs, more attention should be paid to the impact of combined AR or AH on ETD (Supplemental Table 2 summarizes studies on combination therapy in comorbidities).

Research summaries on the mentioned anti-allergic drugs have been compiled in Supplemental Table 1.

### Immunotherapy

Immune modulatory oligonucleotides (IMO) are immunomodulators containing DNA sequences with unmethylated CpG motifs that have the ability to regulate the immune response to allergens.^79^ IMO can activate cell signaling pathways by binding to Toll-like receptor 9, inducing a shift from Th2 to Th1 immune response, resulting in a significant reduction in eosinophil levels, as well as IL-4, IL-5, and IL-13 levels.^80^ Previous studies have shown that IMO is effective in treating and preventing ETD in rats. In ETD rat models induced by OVA stimulation in the middle ear or nasal cavity, pre-treatment of IMO significantly reduced ET passive opening and closing pressures, improved ET ventilation function, and significantly improved mucociliary function compared to the OVA-sensitized control group without IMO pretreatment.^79^, ^81^, ^82^ However, there are currently no clinical studies on this drug, and future research should focus on elucidating the functional differences of this drug in humans and animals, as well as its safety and potential side effects.

In addition, allergen immunotherapy (AIT) is one of the methods for treating allergic diseases. It involves exposing patients to gradually increasing doses of allergens to induce immune tolerance, resulting in significantly reduced or even absent symptoms upon subsequent exposure to the allergen. Currently, AIT has shown good efficacy in treating AR and asthma, and several studies have even demonstrated complete resolution of middle ear effusion and symptoms in most OME patients treated with AIT.^83^^,^^84^ However, there is limited literature on the use of AIT for treating ETD, and existing research is still lacking in terms of sample size, biomarker assessment, and longer-term outcomes. Fortunately, AIT has shown promising results in treating OME, and we hope that future research will provide another effective treatment option for ETD.

### Other treatments

In recent years, multiple studies have confirmed that the use of topical surfactant in animal models of ETD can quickly relieve symptoms and reduce the opening pressure of the ET.^85^, ^86^, ^87^ In a recent phase 2 clinical trial, a surfactant (OP0201) was administered intranasally to young children with AOM for the first time. Although no significant clinical differences were observed between the treatment groups, the safety of OP0201 administration was confirmed, and no adverse effects were reported.^88^

With the gradual exploration in clinical research, surfactant may become an effective treatment for ETD or OME in the future.

In addition, leukotriene antagonists such as montelukast are also considered to be effective in treating allergic OME. Multiple studies have shown that the use of leukotriene antagonists in combination with other drugs leads to a higher rate of OME resolution and is more effective in preventing chronic OME.^89^^,^^90^ Given the relatively safe profile and lack of significant side effects of leukotriene antagonists, they have potential value in the treatment of allergic OME or ETD.

In conclusion, we believe that there is sufficient evidence to support the association between allergic reactions and ETD, and that the impact of allergic reactions should be considered in the treatment and prevention of ETD. However, there are still many problems and challenges in this field. For example, (1) it is still unknown whether ETD is a separate disease, and there is currently no clear gold standard for diagnosis, which leads to bias in the homogeneity of the research subjects in ETD studies. Moreover, since most mechanical obstructions of ET are caused by primary diseases such as AR or AH, it is difficult to distinguish the disease targets when evaluating the efficacy of anti-allergic therapy for this type of ETD, which is one of the reasons why there is no complete treatment plan for ETD at present. (2) Although the concept of a unified airway has been proposed, it is still unclear whether ETD and adjacent allergic diseases occur simultaneously or have a causal relationship in the allergic reactions process. Furthermore, further research is needed to confirm whether the pathological changes and the inflammatory mediators involved in the development of allergic reactions have specificity. (3) Due to the deep location of the ET, complex surrounding structures, and proximity to important blood vessels and nerves, it is difficult to accurately dissect ET mucosal tissue without containing muscle, cartilage, and other surrounding tissue components. This makes traditional biochemical detection methods inaccurate and ineffective for detecting allergic-related immune factors in ET mucosa. Therefore, future research should focus on exploring these issues in depth to provide a more scientific basis for the exact pathogenesis and treatment of ETD.

## Abbreviations

ETD, Eustachian tube dysfunction; ET, Eustachian tube; OME, otitis media with effusion; Th2, T helper 2; OVA, ovalbumin; MCC, Mucociliary Clearance; AOM, acute otitis media with effusion; AR, Allergic Rhinitis; AH, Adenoidal Hypertrophy; LPR, late-phase allergic reactions; INCs, intranasal corticosteroids; VAS, visual analogue scale; ETDQ-7, Eustachian Tube Dysfunction Questionnaire-7; IMO, Immune modulatory oligonucleotides; AIT, allergen immunotherapy.

## Acknowledgments

We are grateful to the Institute of Otorhinolaryngology, Tongji Medical College, Huazhong University of Science and Technology.

## Funding sources

This work was supported by the 10.13039/501100001809National Natural Science Foundation of China [Grant numbers 82071057, 82101229] and Key Research and Development Program of Hubei Province Project [Grant 2021BCA144].

## Availability of data and materials

No datasets were used for the current study.

## Author contributions

All authors contributed substantially to the design of the study, analysis and writing of the article.

## Ethics approval to and consent to participate

This manuscript is a review. It did not involve human or animal subject.

## Consent for publication

The authors' consented to the publication of this review.

## Declaration of competing interest

We declare that we have no conflict of interest.

## Originality statement

We declare that the present manuscript is original and has not been published before nor submitted to another journal for the consideration of publication.

## References

[1] Yang S.M., Hou Z.H., Zhang J. (2018). Expert consensus on Eustachian tube dysfunction. Chin J Otorhinolaryngol Head Neck Surg.

[2] Schilder A.G.M., Bhutta M.F., Butler C.C. (2015). Eustachian tube dysfunction: consensus statement on definition, types, clinical presentation and diagnosis. Clin Otolaryngol Off J ENT-UK Off J Neth Soc Oto-Rhino-Laryngol Cervico-Facial Surg.

[3] Shan A., Ward B.K., Goman A.M. (2019). Prevalence of eustachian tube dysfunction in adults in the United States. JAMA Otolaryngol Neck Surg.

[4] Smith M.E., Bance M.L., Tysome J.R. (2019). Advances in Eustachian tube function testing. World J Otorhinolaryngol - Head Neck Surg.

[5] Lu Y.P., Xu J.T., Liu P. (2020). Advances in the study of Eustachian tube function. Chin J Otol.

[6] Krouse J.H. (2008). The unified airway--conceptual framework. Otolaryngol Clin North Am.

[7] Lemanske R.F., Busse W.W. (1997). Asthma. JAMA.

[8] Thompson H., Tucker A.S. (2013). Dual origin of the epithelium of the mammalian middle ear. Science.

[9] Nguyen L.H.P., Manoukian J.J., Sobol S.E. (2004). Similar allergic inflammation in the middle ear and the upper airway: evidence linking otitis media with effusion to the united airways concept. J Allergy Clin Immunol.

[10] Luong A., Roland P.S. (2008). The link between allergic rhinitis and chronic otitis media with effusion in atopic patients. Otolaryngol Clin North Am.

[11] Ho I.-C., Miaw S.-C. (2016). Regulation of IL-4 expression in immunity and diseases. Adv Exp Med Biol.

[12] Zou Y., Wu H., Zong S.M. (2022). Allergy in the pathogenesis of otitis media with effusion. Chin J Otorhinolaryngol Head Neck Surg.

[13] Pollock H.W., Ebert C.S., Dubin M.G. (2002). The role of soluble interleukin-4 receptor and interleukin-5 antibody in preventing late-phase allergy-induced eustachian tube dysfunction. Otolaryngol--Head Neck Surg Off J Am Acad Otolaryngol-Head Neck Surg.

[14] Liu H., Zhao S.Q., Liu B. (2008). The establishment of the animal model of otitis media with effusion induced by ovalbumin. J Audiol Speech Pathol.

[15] Liu J.Z., jia Kong W. (2008). Dynamic histomorphologic study of otitis media with effusion induced by ovalbumin in rats. Med J Wuhan Univ.

[16] Liu H., Zhao S.Q., Tong J. (2011). A study of Th2/Th1 imbalance in the rat model of otitis media with effusion. Chin J Clin Electron Ed.

[17] Shi C., Zhang H.L., Xu T. (2022). The association between type Ⅰ allergy and otitis media. J Audiol Speech Pathol.

[18] Wright E.D., Hurst D., Miotto D. (2000). Increased expression of major basic protein (MBP) and interleukin-5(IL-5) in middle ear biopsy specimens from atopic patients with persistent otitis media with effusion. Otolaryngol--Head Neck Surg Off J Am Acad Otolaryngol-Head Neck Surg.

[19] Bao K., Reinhardt R.L. (2015). The differential expression of IL-4 and IL-13 and its impact on type-2 immunity. Cytokine.

[20] Kim D.-K., Park H.E., Back S.-A. (2016). Otitis media with effusion in an allergic animal model: a functional and morphological study. Int J Pediatr Otorhinolaryngol.

[21] Shim J.J., Dabbagh K., Ueki I.F. (2001). IL-13 induces mucin production by stimulating epidermal growth factor receptors and by activating neutrophils. Am J Physiol Lung Cell Mol Physiol.

[22] Kerschner J.E., Meyer T.K., Burrows A. (2004). Chinchilla middle ear epithelial mucin gene expression in response to inflammatory cytokines. Arch Otolaryngol Head Neck Surg.

[23] Lin J., Tsuprun V., Kawano H. (2001). Characterization of mucins in human middle ear and Eustachian tube. Am J Physiol Lung Cell Mol Physiol.

[24] Takeuchi K., Maesako K., Yuta A. (1994). Interleukin-8 gene expression in middle ear effusions. Ann Otol Rhinol Laryngol.

[25] Wang W.P., Wang H.T. (2022). Effects of allergy on eustachian tube function and otitis media with effusion. Chin J Otol.

[26] Tanaka A., Ohashi Y., Kakinoki Y. (1998). Influence of the allergic response on the mucociliary system in the eustachian tube. Acta Oto-Laryngol Suppl.

[27] Hardy S.M., Heavner S.B., White D.R. (2001). Late-phase allergy and eustachian tube dysfunction. Otolaryngol--Head Neck Surg Off J Am Acad Otolaryngol-Head Neck Surg.

[28] Oh J.-H., Kim W.J. (2016). Interaction between allergy and middle ear infection. Curr Allergy Asthma Rep.

[29] Smirnova M.G., Birchall J.P., Pearson J.P. (2005). Evidence of T-helper cell 2 cytokine regulation of chronic otitis media with effusion. Acta Otolaryngol (Stockh).

[30] Seong J.-K., Koo J.S., Lee W.J. (2002). Upregulation of MUC8 and downregulation of MUC5AC by inflammatory mediators in human nasal polyps and cultured nasal epithelium. Acta Otolaryngol (Stockh).

[31] Smirnova M.G., Birchall J.P., Pearson J.P. (2004). The immunoregulatory and allergy-associated cytokines in the aetiology of the otitis media with effusion. Mediators Inflamm.

[32] Yang B., Brook C.D. (2017). The role of allergy in otologic disease. Otolaryngol Clin North Am.

[33] Bernstein J.M., Doyle W.J. (1994). Role of Ige-mediated hypersensitivity in otitis media with effusion: pathophysiologic considerations. Ann Otol Rhinol Laryngol.

[34] Guo L.N., Liu W., Zhang J. (2022). The relationship between the surfactant and Eustachian tube function. Int J Otolaryngol Head Neck Surg.

[35] McGuire J.F. (2002). Surfactant in the middle ear and eustachian tube: a review. Int J Pediatr Otorhinolaryngol.

[36] Paananen R., Postle A.D., Clark G. (2002). Eustachian tube surfactant is different from alveolar surfactant: determination of phospholipid composition of porcine eustachian tube lavage fluid. J Lipid Res.

[37] Zhu Z.H., Shan Y.J., Han Y. (2013). Pathological study of otitis media with effusion after treatment with intranasal pulmonary surfactant. Laryngoscope.

[38] Ma Z., Dai C., Yang S. (2007). Protective effect of pulmonary surfactant on cilia of Eustachian tube in otitis media with effusion. Int J Pediatr Otorhinolaryngol.

[39] Abdel-Razek O., Liu T., Chen X. (2021). Role of surfactant protein D in experimental otitis media. J Innate Immun.

[40] Fu J.Y., Chen Y.L., Luo W.S. (2009). Adult allergic rhinitis and eustachian tube and middle ear functions. Chin J Otol.

[41] Ma Y., Liang M., Tian P. (2020). Eustachian tube dysfunction in patients with house dust mite-allergic rhinitis. Clin Transl Allergy.

[42] Juszczak H., Aubin-Pouliot A., Sharon J.D. (2019). Sinonasal risk factors for eustachian tube dysfunction: cross-sectional findings from NHANES 2011-2012. Int Forum Allergy Rhinol.

[43] Yeo S.G., Park D.C., Eun Y.G. (2007). The role of allergic rhinitis in the development of otitis media with effusion: effect on eustachian tube function. Am J Otolaryngol.

[44] Bousquet J., Anto J.M., Bachert C. (2020). Allergic rhinitis. Nat Rev Dis Primer.

[45] Yazıcı H. (2015). Nasal mucociliary clearance in adenoid hypertrophy and otitis media with effusion. Curr Allergy Asthma Rep.

[46] Manno A., Iannella G., Savastano V. (2021). Eustachian tube dysfunction in children with adenoid hypertrophy: the role of adenoidectomy for improving ear ventilation. Ear Nose Throat J.

[47] Skoloudik L., Kalfert D., Valenta T. (2018). Relation between adenoid size and otitis media with effusion. Eur Ann Otorhinolaryngol Head Neck Dis.

[48] Roditi R.E., Shin J.J. (2018). The influence of Age on the relationship between allergic rhinitis and otitis media. Curr Allergy Asthma Rep.

[49] Zhang S.J., Chen X.H. (2013). Relationship between immune resoonse and secretory otitis media. J Clin Otorhinolaryngol Head Neck Surg.

[50] Quaranta N., Milella C., Iannuzzi L. (2013). A study of the role of different forms of chronic rhinitis in the development of otitis media with effusion in children affected by adenoid hypertrophy. Int J Pediatr Otorhinolaryngol.

[51] Fadal R.G. (1993). IgE-mediated hypersensitivity reactions. Otolaryngol--Head Neck Surg Off J Am Acad Otolaryngol-Head Neck Surg.

[52] Norman G., Llewellyn A., Harden M. (2014). Systematic review of the limited evidence base for treatments of Eustachian tube dysfunction: a health technology assessment. Clin Otolaryngol Off J ENT-UK Off J Neth Soc Oto-Rhino-Laryngol Cervico-Facial Surg.

[53] Downs B.W., Butehorn H.F., Prazma J. (2001). Action of histamine on eustachian tube function. Otolaryngol--Head Neck Surg Off J Am Acad Otolaryngol-Head Neck Surg.

[54] Ebert C.S., Pollock H.W., Dubin M.G. (2002). Effect of intranasal histamine challenge on Eustachian tube function. Int J Pediatr Otorhinolaryngol.

[55] Teixeira M.S., Alper C.M., Martin B.S. (2017). Histamine applied topically to the nasal mucosa increases the transmucosal nitrous oxide exchange for the middle ear. Ann Otol Rhinol Laryngol.

[56] Cutler J.L., Labadie R.F. (2008). Effects of ototopical antihistamine on otitis media in an allergic rat. Laryngoscope.

[57] Suzuki M., Kerakawauchi H., Kawauchi H. (1999). Efficacy of an antiallergic drug on otitis media with effusion in association with allergic rhinitis. An experimental study. Ann Otol Rhinol Laryngol.

[58] Labadie R.F., Jewett B.S., Hart C.F. (1999). Allergy increases susceptibility to otitis media with effusion in a rat model. Second place--resident clinical science award 1998. Otolaryngol--Head Neck Surg Off J Am Acad Otolaryngol-Head Neck Surg.

[59] Goodrich T., Rubio F., Cutler J.L. (2009). Otitis media and antihistamines. Curr Allergy Asthma Rep.

[60] Griffin G., Flynn C.A. (2011). Antihistamines and/or decongestants for otitis media with effusion (OME) in children. Cochrane Database Syst Rev.

[61] Chonmaitree T., Saeed K., Uchida T. (2003). A randomized, placebo-controlled trial of the effect of antihistamine or corticosteroid treatment in acute otitis media. J Pediatr.

[62] Griffin G., Flynn C.A., Bailey R.E. (2006). Cochrane Database of Systematic Reviews. (The Cochrane Collaboration.

[63] Roditi R.E., Caradonna D.S., Shin J.J. (2019). The proposed usage of intranasal steroids and antihistamines for otitis media with effusion. Curr Allergy Asthma Rep.

[64] Simpson S.A., Lewis R., van der Voort J. (2011). Oral or topical nasal steroids for hearing loss associated with otitis media with effusion in children. Cochrane Database Syst Rev.

[65] McCoul E.D., Weinreich H.M., Mulder H. (2019). Health care utilization and prescribing patterns for adult eustachian tube dysfunction. Otolaryngol--Head Neck Surg Off J Am Acad Otolaryngol-Head Neck Surg.

[66] Wu A.W., Ow R.A., Tang D.M. (2021). Effect of nasal fluticasone exhalation delivery system on Eustachian tube dysfunction. Int Forum Allergy Rhinol.

[67] Gluth M.B., McDonald D.R., Weaver A.L. (2011). Management of eustachian tube dysfunction with nasal steroid spray: a prospective, randomized, placebo-controlled trial. Arch Otolaryngol Head Neck Surg.

[68] Llewellyn A., Norman G., Harden M. (2014). Interventions for adult Eustachian tube dysfunction: a systematic review. Health Technol Assess Winch Engl.

[69] Mehta N.K., Ma C., Nguyen S.A. (2022). Medical management for eustachian tube dysfunction in adults: a systematic review and meta-analysis. Laryngoscope.

[70] El-Anwar M.W., Nofal A.A.-F., Khazbak A.O. (2015). The efficacy of nasal steroids in treatment of otitis media with effusion: a comparative study. Int Arch Otorhinolaryngol.

[71] Williamson I., Benge S., Barton S. (2009). Topical intranasal corticosteroids in 4-11 year old children with persistent bilateral otitis media with effusion in primary care: double blind randomised placebo controlled trial. BMJ.

[72] Berkman N.D., Wallace I.F., Steiner M.J. (2013). AHRQ Comparative Effectiveness Reviews.

[73] Butler C.C., Van Der Voort J.H. (2002). Oral or topical nasal steroids for hearing loss associated with otitis media with effusion in children. Cochrane Database Syst Rev.

[74] Rosenfeld R.M., Shin J.J., Schwartz S.R. (2016). Clinical practice guideline: otitis media with effusion (update). Otolaryngol--Head Neck Surg Off J Am Acad Otolaryngol-Head Neck Surg.

[75] Crowson M.G., Ryan M.A., Ramprasad V.H. (2017). Intranasal fluticasone associated with delayed tympanostomy tube placement in children with eustachian tube dysfunction. Int J Pediatr Otorhinolaryngol.

[76] Bilgili A.M., Durmaz H.Ö., Dilber M. (2023). Eustachian tube dysfunction in children with adenoid hypertrophy: the effect of intranasal Azelastine-fluticasone spray treatment on middle ear ventilation and adenoid tissue. Ear Nose Throat J.

[77] Bhargava R., Chakravarti A. (2014). A double-blind randomized placebo-controlled trial of topical intranasal mometasone furoate nasal spray in children of adenoidal hypertrophy with otitis media with effusion. Am J Otolaryngol.

[78] Cengel S., Akyol M.U. (2006). The role of topical nasal steroids in the treatment of children with otitis media with effusion and/or adenoid hypertrophy. Int J Pediatr Otorhinolaryngol.

[79] Blanks D.A., Ebert C.S.J. (2008). Immune modulatory oligonucleotides in the prevention and treatment of allergen-induced eustachian tube dysfunction in the animal model. Curr Allergy Asthma Rep.

[80] Blanks D.A., Ebert C.S.J., Eapen R.P. (2007). Immune modulatory oligonucleotides in the prevention and treatment of OVA-induced eustachian tube dysfunction in rats. Otolaryngol--Head Neck Surg Off J Am Acad Otolaryngol-Head Neck Surg.

[81] Ebert C.S.J., Rose A.S., Blanks D.A. (2007). Immune modulatory oligonucleotides in prevention of nasal allergen-induced Eustachian tube dysfunction in rats. Otolaryngol--Head Neck Surg Off J Am Acad Otolaryngol-Head Neck Surg.

[82] Ebert C.S., Rose A.S., Patel M.R. (2006). The role of immunomodulatory oligonucleotides in prevention of OVA-induced Eustachian tube dysfunction. Int J Pediatr Otorhinolaryngol.

[83] Hurst D.S. (2008). Efficacy of allergy immunotherapy as a treatment for patients with chronic otitis media with effusion. Int J Pediatr Otorhinolaryngol.

[84] La Mantia I., Varricchio A., Ciprandi G. (2021). Allergen immunotherapy in children with otitis media with effusion: a preliminary experience. Eur Ann Allergy Clin Immunol.

[85] Venkatayan N., Troublefield Y.L., Connelly P.E. (2000). Intranasal surfactant aerosol therapy for otitis media with effusion. Laryngoscope.

[86] Chandrasekhar S.S., Mautone A.J. (2004). Otitis media: treatment with intranasal aerosolized surfactant. Laryngoscope.

[87] Jang C.H., Park H., Choi C.H. (2010). Efficacy of transnasal nebulized surfactant on experimental otitis media with effusion in Guinea pig. Int J Pediatr Otorhinolaryngol.

[88] Muniz G.B., Shope T.R., Bhatnagar S. (2021). Intranasal surfactant for acute otitis media: a randomized trial. Pediatrics.

[89] Ertugay C.K., Cingi C., Yaz A. (2013). Effect of combination of montelukast and levocetirizine on otitis media with effusion: a prospective, placebo-controlled trial. Acta Otolaryngol (Stockh).

[90] Nakamura Y., Hamajima Y., Suzuki M. (2016). The effect of the leukotriene antagonist pranlukast on pediatric acute otitis media. Int J Pediatr Otorhinolaryngol.

