# Supplementary material for: Allergy in pathogenesis of Eustachian Tube Dysfunction
Source: World Allergy Organ J. 2024 Jan 5;17(1):100860. doi: 10.1016/j.waojou.2023.100860 (PMC10809091; doi:10.1016/j.waojou.2023.100860)
Supplement: Multimedia component 1 [file mmc1.docx]

**Supplemental Figure**

**
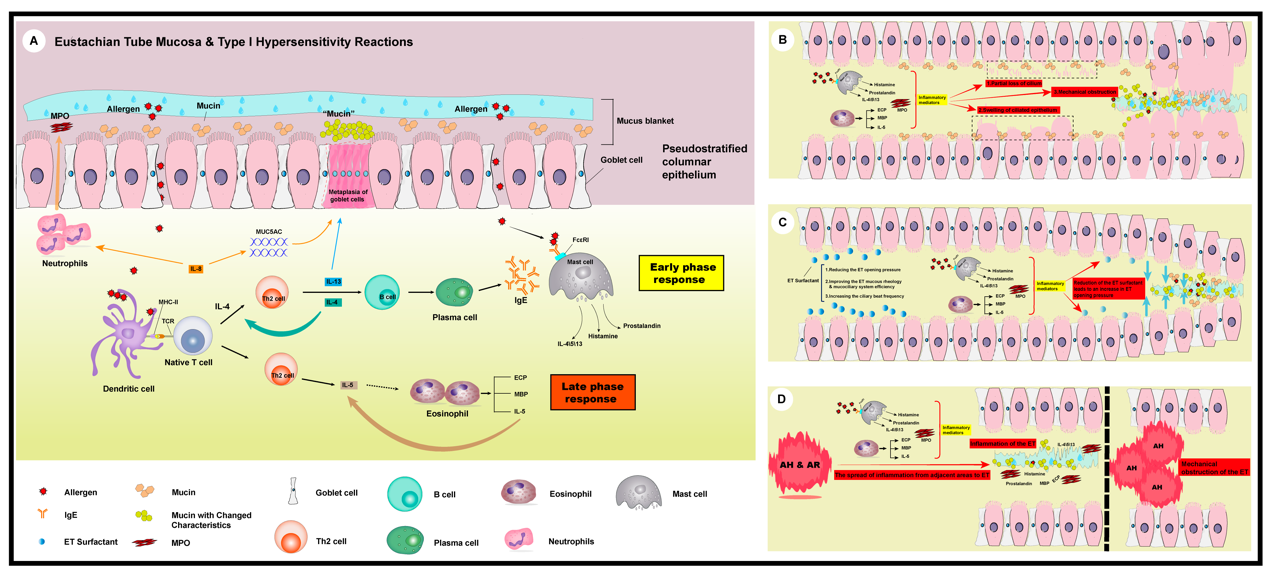
**

**Supporting information legends:**

Fig. 1: The Role of Allergic Reactions in the Pathogenesis of Eustachian Tube Disfunction. (A) The classic type I allergic reactions mechanism includes early and late-phase allergic reactions. It involves immune regulation mainly by T helper 2 (Th2) cytokines, which may play a possible role in ETD. (B) Accumulation of allergic inflammatory mediators can lead to the partial loss of cilium in the ET mucosa, swelling of the ciliated epithelium, and mechanical obstruction, resulting in ETD. (C) When the ET becomes inflamed or suffers from inflammation, its surfactant can be damaged, leading to impaired active opening function and causing ETD. (D) Allergic diseases in the adjacent areas of the ET, such as AR or AH, can directly spread to the ET, causing the accumulation of inflammatory mediators and triggering the process described in Fig. 1B above. In addition, AH can also directly cause mechanical obstruction of the ET orifice. MPO: myeloperoxidase; ECP: eosinophil cationic protein; MBP: major basic protein; FcεRI: high affinity receptors for IgE; ETD: Eustachian tube disfunction; ET: Eustachian tube; AR: allergic rhinitis; AH: adenoidal hypertrophy; Supplemental Table 2
