# Supplementary material for: Allergy in pathogenesis of Eustachian Tube Dysfunction
Source: World Allergy Organ J. 2024 Jan 5;17(1):100860. doi: 10.1016/j.waojou.2023.100860 (PMC10809091; doi:10.1016/j.waojou.2023.100860)
Supplement: Multimedia component 2 [file mmc2.docx]

**Table 1 Study on the therapeutic effect of anti-allergic drugs on ETD**

| **Types** | **Authors** | **Year** | **Study**  **Design** | | **Specific**  **Drugs** | **Research**  **Diseases** | **Evaluation** | **Y/N** |
| --- | --- | --- | --- | --- | --- | --- | --- | --- |
| **Antihistamines** | Chonmaitree et al. | 2003 | Prospective double-blinded placebo-controlled RCT | Chlorpheniramine maleate | | AOM | Antihistamines may inhibit ET function. | N |
|  | Griffin et al. | 2006 /2011 | systematic  review |  | | OME | Antihistamines, decongestants, or their combination have no benefit in preventing or treating OME, and the incidence of side effects is about 10%. | N |
|  | Roditi et al. | 2019 | systematic  review |  | | OME | Antihistamines cannot achieve long-term improvement in OME. | N |
|  | **Using in ETD:** | | | | | | |  |
|  | Gluth et al. | 2011 | Prospective double-blinded placebo-controlled RCT | Intranasal Aqueous Triamcinolone acetonide | | ETD | The use of INCs is not effective in treating ETD. | N |
|  | Llewellyn et al. | 2014 | Systematic  review |  | | ETD | No improvement in symptoms or middle ear function observed in patients with otitis media with effusion and/or negative middle ear pressure. | N |
|  | Wu et al. | 2020 | A retrospective, cohort study | Xhance nasal spray | | ETD | The use of novel fluticasone propionate exhalation delivery system can improve symptoms in the majority of chronic ETD patients. | Y |
|  | Mehta et al. | 2022 | Systematic  review |  | | ETD | INCS are ineffective for chronic ETD. | N |
|  | **Using in OME:** | | | | | | |  |
| **INCs** | El-Anwar et al. | 2015 | Prospective single blinded | Momentasone furoate spray | | OME | INCs can effectively treat OME. | Y |
|  | Wiliamson et al. | 2009 | Prospective double-blinded placebo-controlled RCT | Mometasone furoate spray | | OME | INCs has no significant therapeutic effect on bilateral secretory otitis media in children. | N |
|  | Simpson et al. | 2011 | Systematic  review |  | | OME | Lack of evidence for long-term or short-term benefits of INCs for OME effusion. | N |
|  | Berkman et al. | 2013 | Systematic  review |  | | OME | When used alone or in combination with antibiotics, INCs did not show a difference in cure rate for OME. | N |
|  | Rosenfeld et al. | 2016 | *Clinical Practice Guideline: Otitis Media with Effusion* |  | | OME | Opposition to the use of INCs for treating OME. | N |
|  | Roditi et al. | 2019 | systematic  review |  | | OME | INCs cannot improve middle ear effusion in patients with OME in the long term. | N |
| **AIT** | Hurst et al. | 2008 | A prospective, cohort study |  | | OME | Effectively prevent or limit the duration of OME. | Y |
|  | La Mantia et al. | 2021 | A prospective, open study |  | | OME | More than half of the children experienced complete disappearance of OME after AIT. | Y |
| **Leukotriene**  **antagonists** | Ertugay et al. | 2013 | Prospective double-blinded placebo-controlled RCT | Montelukast & levocetirizine | | OME | Combination of two drugs may have a beneficial effect on clinical improvement of middle ear fluid in children with OME. | Y |
|  | Nakamura et al. | 2016 | Prospective RCT | Pranlukast & antibiotics | | AOM | Combining pranlukast with antibiotics decreases the frequency of persistent OME following AOM in children. | Y |

**Table 1：****Study on the therapeutic effect of anti-allergic drugs on ETD**. ETD, Eustachian tube dysfunction; ET, Eustachian tube; OME, otitis media with effusion; AOM, acute otitis media with effusion; INCs, intranasal corticosteroids; RCT, Randomized controlled trial; Y: supporting use; N: against use; AIT: allergen immunotherapy.
