# Supplementary material for: Allergy in pathogenesis of Eustachian Tube Dysfunction
Source: World Allergy Organ J. 2024 Jan 5;17(1):100860. doi: 10.1016/j.waojou.2023.100860 (PMC10809091; doi:10.1016/j.waojou.2023.100860)
Supplement: Multimedia component 3 [file mmc3.docx]

**Table 2** **Treatment of Comorbidities with Individual or Combined Use of INCs**

| **Authors** | **Year** | **Study Design** | **Types of**  **Drugs** | **Research**  **Diseases** | **Evaluation** |
| --- | --- | --- | --- | --- | --- |
| Crowson et al. | 2017 | A retrospective study | INCs | OME&ETD | INCs may reduce the probability of tympanostomy tube placement. |
| Yang SM et al. | 2018 | *the Chinese Consensus on Eustachian Tube Dysfunction* | INCs | AR&ETD | INCs can be given to ETD patients with AR |
| Ma Y et al. | 2020 | Prospective controlled cross-sectional study | INCs & antihistamine | AR&ETD | The combined use of INCs and antihistamines can improve nasal symptoms and ET function. |
| Bilgili et al. | 2023 | Prospective randomized longitudinal study | INCs & antihistamine | AH&ETD | Combined therapy significantly improves adenoid tissue atrophy and ETD. |
| Cengel et al. | 2006 | Prospective RCT | INCs | AH&OME | INCs obviously alleviates the symptoms of AH and OME. For OME, it is a useful alternative to surgery in the short term. |
| Bhargava et al. | 2014 | Prospective double-blinded placebo-controlled RCT | INCs | AH&OME | INCs therapy is effective in treating patients with adenoid hypertrophy and secretory otitis media. |

**Table 2: Combination therapy or treatment for comorbidities**. RCT, Randomized controlled trial; ETD, Eustachian tube dysfunction; OME, otitis media with effusion; INCs, intranasal corticosteroids; AR, Allergic Rhinitis; AH, Adenoidal Hypertrophy;
